# Successful outcome of disseminated mucormycosis in a 3-year-old child suffering from acute leukaemia: the role of isavuconazole? A case report

**DOI:** 10.1186/s40360-018-0273-7

**Published:** 2018-12-06

**Authors:** Marjorie Cornu, Bénédicte Bruno, Séverine Loridant, Pauline Navarin, Nadine François, Fanny Lanternier, Elisa Amzallag-Bellenger, François Dubos, Françoise Mazingue, Boualem Sendid

**Affiliations:** 1 Laboratoire de Parasitologie Mycologie, CHU Lille, Univ. Lille, INSERM U995 – LIRIC - Lille Inflammation Research International Centre, F-59000 Lille, France; 2 Service d’Onco-Hématologie Pédiatrique, CHU Lille, F-59000 Lille, France; 3 Paris Descartes University, Sorbonne Paris Cité, Infectious Diseases Unit, Necker-Enfants Malades University Hospital, AP-HP, Imagine Institute, Paris, France; 4 Institut Pasteur, Unité de Mycologie Moléculaire, CNRS URA3012, Paris, France; 5 Institut Pasteur, Centre National de Référence Mycoses Invasives et Antifongiques, Paris, France; 6 Service de Radio-pédiatrie, CHU Lille, F-59000 Lille, France; 7 Service des urgences et maladies infectieuses pédiatriques, CHU Lille et Univ. Lille, F-59000 Lille, France

**Keywords:** Isavuconazole, Mucormycosis, Paediatrics, Drug-monitoring, *Lichtheimia*

## Abstract

**Background:**

The use of isavuconazole is approved for the management of invasive aspergillosis and mucormycosis, only in adults, as no paediatric pharmacology studies have been reported to date. Very few paediatric cases have been published concerning the use of isavuconazole. Amphotericin B is the only antifungal agent recommended in paediatric mucormycosis, but adverse effects and especially nephrotoxicity, even with the liposomal formulation, could be problematic. In this context, the use of other antifungal molecules active on Mucorales becomes needful.

**Case presentation:**

We describe a case of mucormycosis with rapid onset dissemination in a 3-year-old girl recently diagnosed with acute lymphocytic leukaemia. She was successfully treated with isavuconazole alone and then in combination with liposomal amphotericin B. Isavuconazole therapy was guided by therapeutic drug monitoring.

**Conclusions:**

This case offers new perspectives on the potential use of isavuconazole in children with mucormycosis, as an alternative or adjunct to liposomal amphotericin B.

## Background

The use of isavuconazole is approved for the management of invasive aspergillosis and mucormycosis, only in adults, as no paediatric pharmacology studies have been reported to date [1]. Very few paediatric cases have been published concerning the use of isavuconazole [2, 3]. Amphotericin B and posaconazole are the only antifungal molecules recommended in mucormycosis in patients with haematological malignancies [4]. However, posaconazole is not yet approved in paediatric population, and the availability of IV and new per os formulations is recent. Moreover adverse effects, especially nephrotoxicity, related to the use of amphotericin B, liposomal formulation included, could be problematic. In children, therapeutic options are limited and the use of other antifungal molecules active on Mucorales becomes necessary.

### Case presentation

At the end of 2015, a 3-year-old girl, with B-cell acute lymphocytic leukaemia with TEL-AML1 fusion and profound neutropenia, was started treatment with EORTC 58081 protocol (NCT01185886), medium risk AR1. After 19 days of prednisolone (60 mg/m^2^/day), two doses of vincristine (1.5 mg/m^2^), one dose of daunorubicin (30 mg/m^2^) and one dose of asparaginase (10,000 IU/m^2^), she developed febrile neutropenia with digestive problems. Procalcitonin and serum C-reactive protein (CRP) levels were 1.85 ng/mL and 91 mg/L, respectively. Chemotherapy was discontinued and antibiotherapy was started with ceftriaxone. Due to persistence of fever, ceftriaxone was switched on Day 3 to piperacillin-tazobactam for 14 days and amikacin for 3 days. At that time, neutrophil count was 0.5 × 10^9^/L. Increasing CRP (350 mg/L) prompted the addition of caspofungin on Day 6 (70 mg/m^2^ day 1, followed by 50 mg/m^2^/day) following a local protocol established according to international guidelines [5]. Twice weekly screening for serum mannan, galactomannan (Platelia™; Bio-Rad Laboratories) and (1,3)-β-D-glucan (Fungitell™; Associates of Cape Cod, Inc.) remained negative. On Day 10 of fever, a chest computed tomography (CT) scan showed condensation in the left lower lobe associated with right pleural effusion evocative of invasive aspergillosis. Treatment with intravenous voriconazole was started (9 mg/kg bid day 1, then 8 mg/kg bid) and caspofungin was stopped. An abdominal ultrasound showed bilateral nephromegaly. Direct microscopic examination, culture, galactomannan detection and *Aspergillus* q-PCR in bronchoalveolar lavage were negative. All blood cultures remained sterile.

**Fig. 1:**
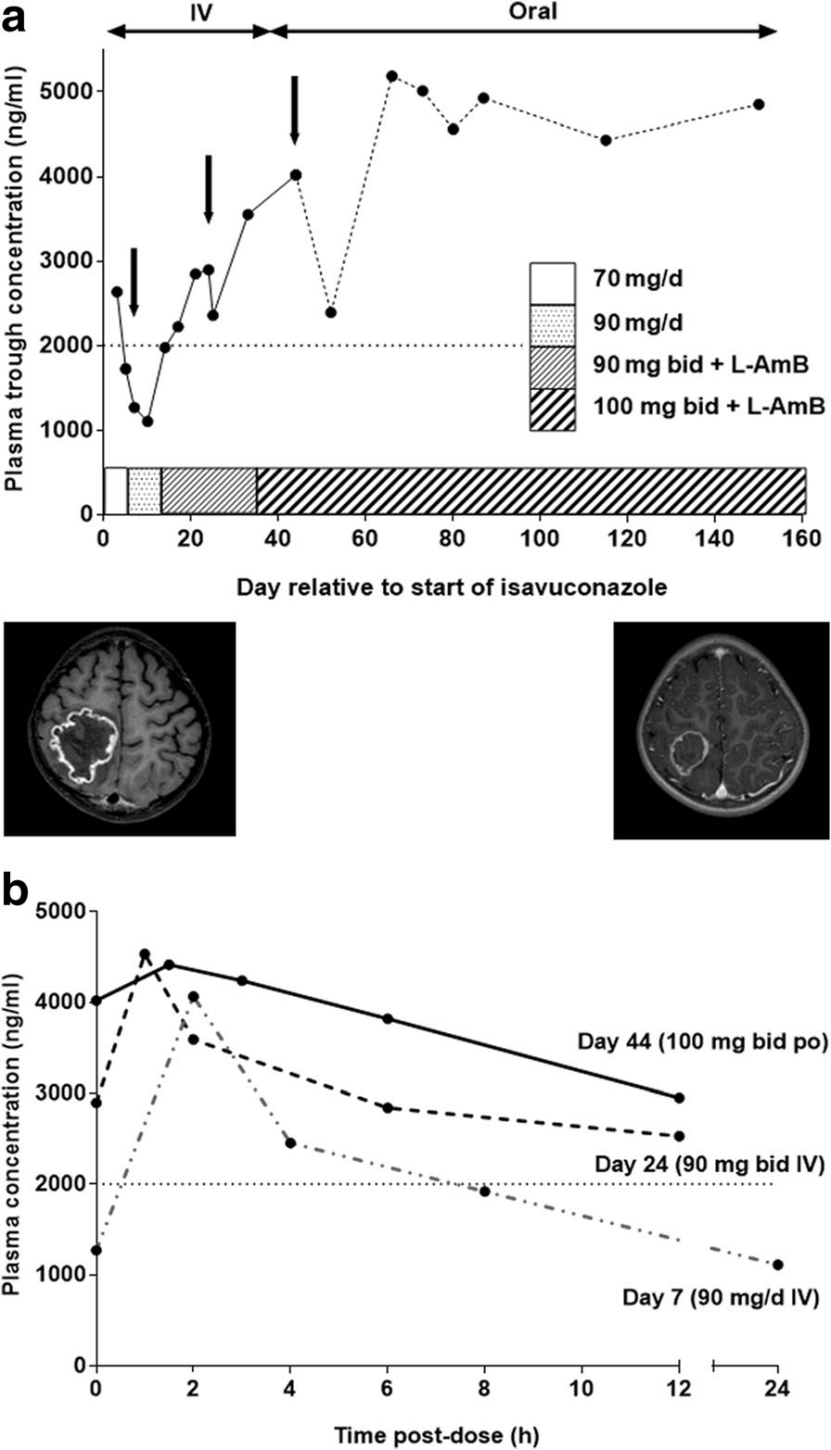
**a** Plasma trough concentration of isavuconazole after intravenous (IV) or oral dosing (arrows indicate times of pharmacokinetic study days). In parallel, cerebral MRIs on Day 21 of fever and after 6 months of antifungal treatment showing regression of a ring-enhanced cerebral lesion (from 5.5 cm in diameter to 3.2 cm) in the right frontal parietal region. **b** Pharmacokinetic profiles of isavuconazole. Plasma concentrations of isavuconazole during 24 h following dosing at day 7 (90 mg/d, IV), and during 12 h following dosing day 24 (90 mg bid, IV) and day 44 (100 mg bid, oral). (bid, twice a day; IV, intravenous; L-AmB, liposomal amphotericin B; po, per os)

On Day 21 of fever, neutrophil count was 11.2 × 10^9^/L. Fundoscopy was performed because of a reduction in visual acuity, revealing multiple subretinal foci suspected to be fungal in origin. Direct microscopy of vitreous humour revealed large, aseptate, ribbon-like hyphae. Among the different mycological techniques used, only Mucorales q-PCR was positive for *Lichtheimia* spp. (Cq 31) in vitreous humour, but was negative in serum. Magnetic resonance imaging (MRI) showed two abscess-like cerebral lesions and confirmed the kidney infiltration (Fig. 1a). A diagnosis of disseminated mucormycosis was established.

Despite limited experience in children, voriconazole was switched to isavuconazole (ISAV) (compassionate off-label use) instead of liposomal amphotericin B (L-AmB), because of renal impairment. Each dose (70 mg every 8 h for 48 h, then 70 mg/day IV) was infused over 1 h. Plasma drug monitoring was implemented from Day 3 after ISAV introduction (AISAVI) and was repeated regularly up to Day 150 (Fig. 1a). Considering a target trough plasma level (TPL) of 2000–4000 ng/mL (approximate average adult TPL observed in Phase 3 studies) [6, 7], low TPL on Day 5 AISAVI prompted an increase in dose to 90 mg/day. A pharmacokinetic profile (Fig. 1b) obtained on Day 7 AISAVI showed that TPL decreased to 1110 ng/mL at 24 h. The ISAV dosage was changed to 90 mg twice daily and L-AmB (10 mg/kg/day) was added despite renal impairment because of the cerebral lesions and difficulties in achieving the ISAV TPL target. The TPL target was reached on Day 17 AISAVI and levels were stable by Day 24. In order to facilitate home nursing care, the route of administration was changed to capsules (100 mg bid). Due to difficulties in swallowing, the capsules were opened and the contents dissolved in an acidic beverage. After 1 week, the mixture was introduced via a nasogastric tube because of vomiting. Median TPL was 4890 ng/mL from Day 66 AISAVI and remained stable over time, while the maximum concentration was obtained between 1 or 2 h after administration, with values varying between 4200 and 4690 ng/ml (Fig. 1b).

With the exception of slight nausea/vomiting during the first 2 months after initiation, no adverse effects were noted with ISAV, and liver enzymes were in the range of normal values observed in healthy subjects. The patient was in complete cytological and molecular remission and was chemotherapy-free for 7 months, until her leukaemia relapsed at 8 months. The patient was treated with the first-line CAALL-F01 protocol (NCT02716233), medium risk, and was in complete remission 4 months after relapse. Because of the risk of drug interactions, L-AmB (7 mg/kg/day) was continued alone for 4 months and was then replaced with ISAV (50 mg twice daily). Antifungal treatment was maintained during maintenance chemotherapy. The size of the cerebral abscesses decreased between the first and last MRI (at 16 months) from 55 × 37 and 45 × 38.4 mm to 23.5 × 18 and 17 × 17 mm with persistence of hypometabolism on a fluorodeoxyglucose-positron emission tomography scan (FDG PET/CT). Renal insufficiency was stable with clearance of 78 ml/min/m^2^ according to the Schwartz formula. The loss of left-sided vision was irreversible. After 24 months of treatment, the patient was still alive without further damage. The treatment timeline is shown in the Table 1.

**Table 1:** Timeline of events

	Clinical features	Biology results	Antimicrobial therapy	
D-19	Start of chemotherapy	Neutropenia		
D0	Febrile neutropenia	High level of CRP and PCT	Antibiotherapy introduction	Ceftriaxone 100 mg/kg/d
D3	Persistence of fever	Rising of CRP and PCT rates	Antibiotherapy switch	Tazobactam Piperacillin 400 mg/kg/d Amikacine
D6	Persistence of fever		Adding antifungal therapy	Caspofungin 70 mg/m^2^ day 1, then 50 mg/m^2^/d
D10	Abnormal chest CT scan and abdominal ultra-sound		Antifungal combined therapy	Adding voriconazole 9 mg/kg bid day 1, then 8 mg/kg bid IV
D16		Negative BAL	Withdrawal Antibiotherapy and caspofungin	
D21		Positive Fundoscopy	Voriconazole intravitreal injection 50 μg/ml	
D24	Abnormal brain MRI Initiation of TDM on D27		Switch antifungal therapy	Isavuconazole 70 mg every 8 h for 48 h, then 70 mg/d IV
D31				Isavuconazole 90 mg/d IV
D37			Antifungal combined therapy	Isavuconazole 90 mg bid IV L-AmB 10 mg/kg/d
D39	Fever resolution			
D58				Isavuconazole 100 mg bid *p.o*
D63	TPL steady state			
M6	Regression of lesions on imagery			
M8	Leukaemia relapse		Withdrawal isavuconazole	L-AmB 7 mg/kg/d
M12	Complete remission		Switch antifungal therapy	Isavuconazole 50 mg bid *p.o*
M16	Regression of lesions on imagery			

*BAL* bronchoalveolar lavage, bid, twice a day, *CRP* C-Reactive protein, *d* day, *IV* intra-venous, *L-AmB* liposomal amphotericin B, *PCT* procalcitonin, *p.o* per os, *TDM* therapeutic drug monitoring, *TPL* trough plasma level

## Discussion and conclusions

The incidence of mucormycosis in Europe is increasing [8–10]. Haematological malignancy is the prominent underlying disease, accounting for 32–38% of cases [10]. Diagnosis of mucormycosis depends on a combination of clinical, radiological and mycological criteria, and is often missed or delayed. Our patient was diagnosed 25 days after the onset of fever. The time between the first symptoms and diagnosis ranges from 0 to 30 weeks [11]. In our patient, disseminated mucormycosis appeared as an early complication of leukaemia (19 days after the start of chemotherapy). However, her fungal infection may have begun to develop before the diagnosis of leukaemia, probably in relation to the initial profound neutropenia. She rapidly presented with cerebral, ocular, pulmonary, muscular and renal lesions. Although surgery may improve survival [12, 13], the discovery of cerebral and disseminated lesions in this patient prevented surgical intervention. L-AmB is the most effective antifungal agent against Mucorales with doses up to 10 mg/kg [12, 14]. More recently, two azoles with potent activity against Mucorales have been developed as alternatives to L-AmB: posaconazole and ISAV [6, 12]. In animal models, azoles penetrate brain tissue well [15]. However, neither posaconazole nor ISAV drug are currently approved for paediatric use. Only an oral suspension posaconazole has been tested in children and this resulted in high inter- and intra-patient variability in serum concentrations [16]. An intravenous formulation was not available in France at that time. ECIL-6 guidelines recommend posaconazole with grade CIII as an alternative treatment if AmB formulations are contraindicated [4]. Recently, two trials (VITAL, SECURE) have been reported which led to the recent indication, in adults, of ISAV use in cases where L-AmB is inappropriate [17]. In view of the renal impairment in our patient, ISAV was considered as a treatment option [18].

According to a paediatric epidemiological study based on two large international registries of mucormycosis, mortality rates range from 41.3 to 66.6% in children suffering from malignancies [13]. Although dissemination was one of two significant factors influencing mortality, our patient is still alive 24 months after the diagnosis of mucormycosis. However, an assessment of the specific contribution of ISAV to this favourable outcome remains difficult. Indeed, the early termination of chemotherapy contributing to faster aplasia recovery, the prolonged remission of leukaemia without chemotherapy and the concomitant use of L-AmB at the start of antifungal therapy may all have played an important role in the outcome. However, it should be emphasized that the fungal lesions continued to regress under ISAV alone. Moreover, tolerance of ISAV was good and this case provides interesting information about ISAV pharmacokinetics and alternative routes of administration in the paediatric population.

Recently, the use of ISAV in three young children, between 4.5 and 7 years of age having developed mucormycosis, has been reported [2, 3]. For two of them, the initial dose of ISAV was lower than that in adult, 80 and 100 mg/day, respectively. The remaining one received the adult dosage (200 mg/day). In the present case, we started with the recommended dose of 70 mg/day after a loading dose. Because of the absence of data in children, initial estimation of dose was obtained via extrapolation approach based on pharmacokinetic-pharmacodynamic modelling and calculation based on body surface area. Altogether, pharmacokinetics studies obtained from these patients suggest that a lower dosage of ISAV is not appropriate for the management of paediatric patients. Indeed, higher drug clearance rate and shorter half- life of ISAV in children as compared with adults may explain lower trough levels obtained in these three cases [2]. The final dosage for two paediatric cases was 200 mg/day, the 7-year-old girl received a higher dose increased to 2 × 200 mg/day. In the three previous cases, while the infection was progressing despite surgery/debridement and lipid formulations of AmB, the clinical states improved with the addition of ISAV as salvage therapy. No side effects were reported in all of these paediatric cases, which underline the safety of isavuconazole in this population.

Although more evidence is needed to support its paediatric use, this case offers new perspectives on the use of ISAV. Due to the current lack of an established paediatric dose, therapeutic drug monitoring of ISAV must be performed to guide therapy.

## Abbreviations

AISAVI: After isavuconazole introduction
CRP: C-reactive protein
FDG PET/CT: Fluorodeoxyglucose-positron emission tomography scan
ISAV: Isavuconazole
L-AmB: Liposomal amphotericin B
MRI: Magnetic resonance imaging
TPL: Trough plasma level
